# Successful treatment of dens invaginatus type 3 with infected invagination, vital pulp, and cystic lession: A case report

**DOI:** 10.1002/ccr3.1647

**Published:** 2018-06-25

**Authors:** Ausra Dembinskaite, Rita Veberiene, Vita Machiulskiene

**Affiliations:** ^1^ Clinic of Dental and Oral Pathology Faculty of Odontology Lithuanian University of Health Sciences Kaunas Lithuania

**Keywords:** cone beam computer tomography, endodontic treatment, large cyst‐like periapical lesions, type 3 dens invaginatus, vital pulp

## Abstract

All efforts should be aimed to safe permanent tooth for adolescents. Presented rare case confirms a possibility to save a tooth with Oehlers Type 3 anomaly with peri‐invaginated periodontitis and to preserve vitality of the tooth pulp, even when surgical cyst removal is performed.

## 1 INTRODUCTION

Dens invaginatus (DI) is a malformation of the tooth. It manifests as deepening or invagination of the enamel organ into the dental papilla prior to calcification of the dental tissues.^1^

Several explanations of this phenomenon have been published; however, the etiology of dens invaginatus remains unclear. DI might be caused by a focal failure of growth of the internal enamel epithelium leading to proliferation of the surrounding normal epithelium with eventual engulfment of the static area. According to Oehler,^2^ distortion of the enamel organ occurs during the tooth development and results in protrusion of a part of the enamel organ. Furthermore, infection, trauma, and genetics have been suggested as possible contributing factors.^3^

The prevalence of DI is reported being between 0.3% and 10%.^1^ The most commonly affected tooth is the maxillary lateral incisor followed by the maxillary central incisor.^1^, ^3^, ^4^

Oehlers^2^ was the first who described different types of dens invaginatus, and categorized them according to the depth of the invagination into the root. Thus, Type 1 invaginations are confined to the crown, Type 2 invaginations extend into the root, but do not penetrate the periodontum and Type 3 invaginations extend into the root and exit laterally (type A) or apically (type B). Usually, no communication with the pulp is present.

The morphology of the crown and the root canal is often complicated. 2D radiographs may not produce sufficient diagnostic information about complexity of the dens invaginatus anatomy. Cone‐beam computed tomographic (CBCT) imaging allows visualization of the third dimension and eliminates superimpositions.^5^, ^6^ Thus, diagnosis and treatment planning of dens invaginatus can be very challenging for a clinician.

Any communication between the oral cavity and the invaginatus foramen can lead to an inflammatory response within the periodontal tissues. The disease is called “peri‐invagination periodontitis” (PIP). Where PIP exists along with the healthy pulp, all efforts should aim to preserve the vitality of the pulp. The objective should therefore be to treat the invagination in isolation of the root canal where possible.^7^

The purpose of this article was to describe the successful case of endodontic and surgical treatment of a maxillary right incisor diagnosed with infected invagination (Oehlers' type 3) associated with apical cyst‐like bone destruction while maintaining vitality of the surrounding pulp.

## 2 CASE REPORT

A 14‐year‐old male patient was referred to the Clinic of Dental and Oral Pathology at Lithuanian University of Health Science, Kaunas, Lithuania, by his general dentist for evaluation and treatment of the maxillary lateral right incisor (tooth #12). The patient complained about constant mild pain, tenderness to bitting and touching mucosa near the tooth #12. He reported swelling history in the infraorbital region about a month ago.

**Figure 1:**
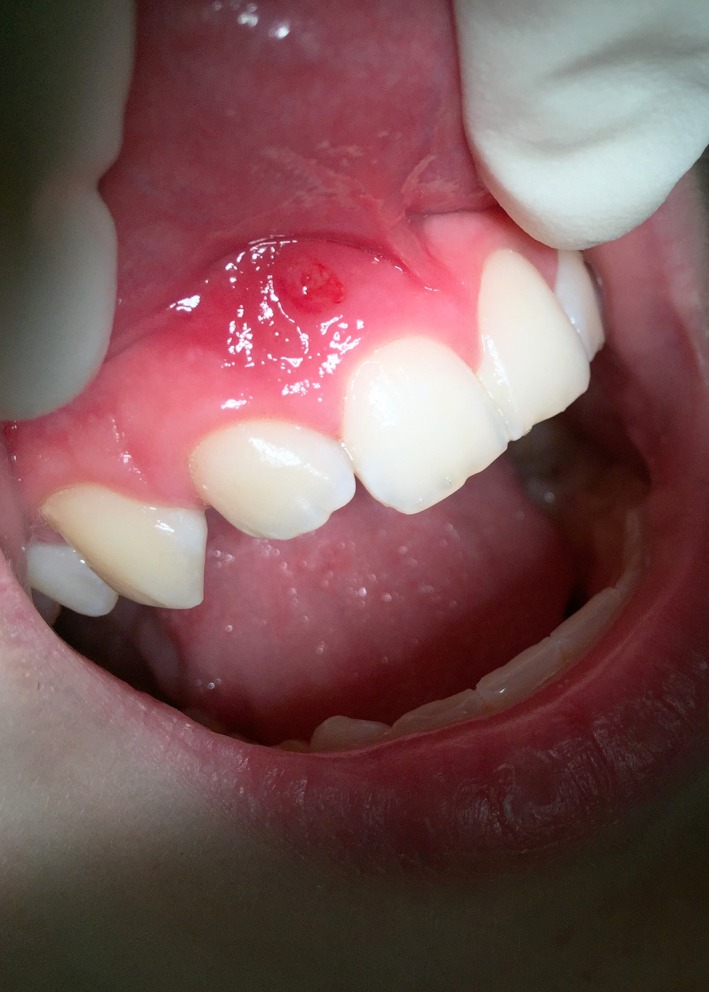
Preoperative photography of the clinical case. Note the position of the maxillary right lateral incisor and the sinus tract in the alveolar mucosa

On the appointment day, the extra‐oral examination revealed no abnormalities. The intraoral examination showed infra‐occlusion of tooth #12. Sinus tract was observed in the alveolar mucosa, proximally to the apical area of tooth #12. The tooth reacted normally to percussion, however, was somewhat sensitive to palpation. Response to cold test was positive, indicating the unaffected vitality of the pulp. There was no evidence of caries in the hard tissues as well as no increased tooth mobility and probing depths (Figure 1).

**Figure 2:**
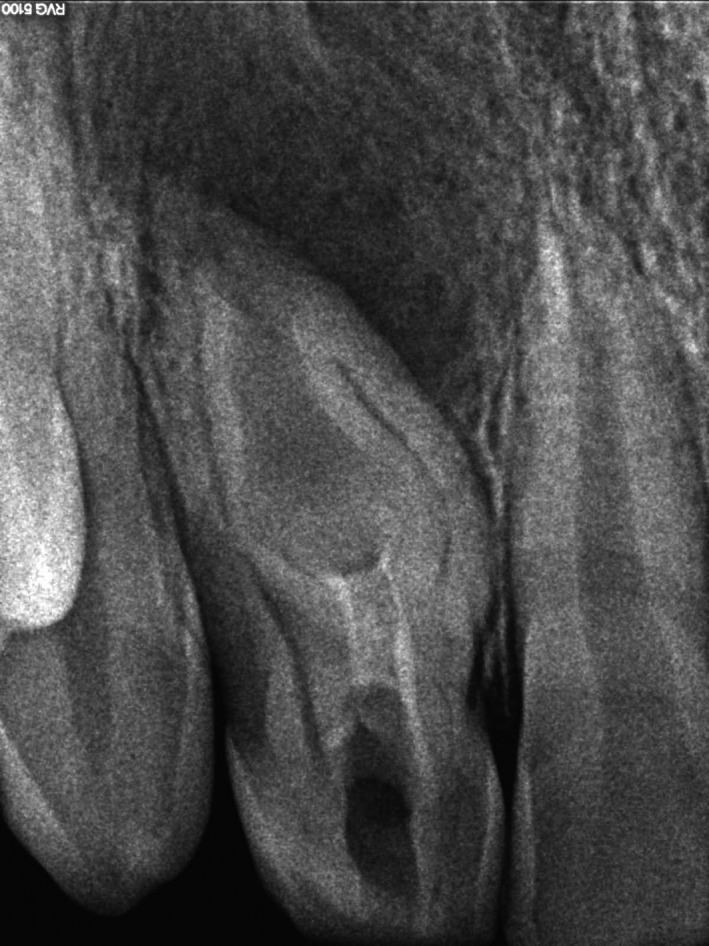
Preoperative radiography of the tooth #12. Note a huge radiolucent lesion in the periapical region and the invagination extending from the crown to the root apex

The tooth presented with an open and unsealed endodontic cavity. Drainage of pus through the cavity was observed. The radiographic examination revealed a huge radiolucent lesion in the periapical region of tooth #12 and signs of a Oehlers' type 3 invagination (Figure 2). The invagination extended from the crown to the root apex (“pseudocanal”), and apparently did not communicate with the regular canal.

**Figure 3:**
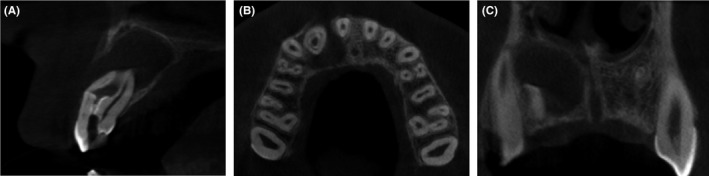
Preoperative cone‐beam computed tomographic (CBCT) presentation of the case. A, Saggital section. Note the large space of the invagination and the extremely compressed pulp space in the buccal side of the root and the presence of a large periapical radiolucency related to the tooth #12. Destruction had no connection with maxillary sinus or the nasal cavity but did penetrate the buccal wall. There was still viable a little of undamage bone structure around the real root canal space in the periapical area. B, Axial section. The invagination was separated from a circular real root canal and had a different portal of exit. C, Coronal section. The shape of radiolucency was quite oval, and it had a quite well circumscribed sclerotic border

With the consent of the patient's mother, a cone beam computer tomography (CBCT) scan (Picasso‐Trio, Vatech Global) with exposure parameters of 80 kVp, 5 mA, and 12‐24 seconds was taken of the area of interest. The CBCT scan revealed the presence of a large periapical radiolucency related to the tooth #12. Radiolucency had a quite well‐circumscribed sclerotic border and no connection with maxillary sinus or the nasal cavity. Coronal and axial CBCT images showed that the invagination was separated from a circular vital root canal and had a different portal of exit (Figure 3).

The final diagnosis was defined as chronical apical periodontitis with a cyst‐like lesion of tooth #12 with an infected Oehlers' type 3 invagination and the surrounding vital pulp. The treatment plan was to perform endodontic treatment of the invagination and then to enucleate the cystic lesion surgically, while maintaining the pulp vitality.

On the first visit, the endodontic cavity of tooth #12 was carefully reshaped without penetrating the pulp using local anesthesia (1.7 mL Ubistesin forte, articaine, 1:100 000 epinephrine) and rubber dam isolation. Under the dental operating microscope (OPMI pico, Carl Zeiss, Jena, Germany), a wide infected invagination in the middle of the cavity was observed. It was not possible to visualize the end of invagination because of the curvature. The pulp horns were hidden behind a thin layer of dentin and located in the mesial and distal corners of the cavity.

The space of invagination was instrumented with K‐files (Dentsply Maillefer, Ballaigues, Switzerland) and generously irrigated with 2.5% NaOCl and 17% EDTA, using an ultrasound activation. Calcium hydroxide paste was inserted for 2 weeks, and the cavity was sealed with temporary material (IRM, Dentsply Maillefer).

On the second appointment, the patient had no complaints, and the sinus tract was closed. The tooth was anesthetized as previously described and isolated with a rubber dam. Then, the apical entrance of the invagination was sealed with mineral trioxide aggregate (MTA, Angellus, Lordina, Brazil) and condensed with hand pluggers and paper points.

After allowing 20 min for setting of MTA, the remaining space of invagination was filled with thermoplastic gutta‐percha (Callamus Dual, Dentsply Mailefer) and AHplus sealer (Dentsply, De Trey, GmbH, Konstanz, Germany). Furthermore, the dentin walls near the vital pulp were covered with “Biodentine” (Septodont, Saint Maur des Fosses, France), and the final composite restoration was placed (3M, Filtek Supreme).

In 3 months, the surgical enucleation of the cyst was performed. Under the dental operating microscope, using intrasulcular incision and vertical release, a full‐thickness flap was reflected and thin cortical bone was removed to create the bony window. The cystic lining/granulation tissue was carefully enucleated, without damaging the periapical region of teeth #11, #12, #13. The integrity of maxillary sinus and nasal cavity was not damaged. The histological evaluation of the biopsy confirmed the diagnosis of a radicular cyst.

**Figure 4:**
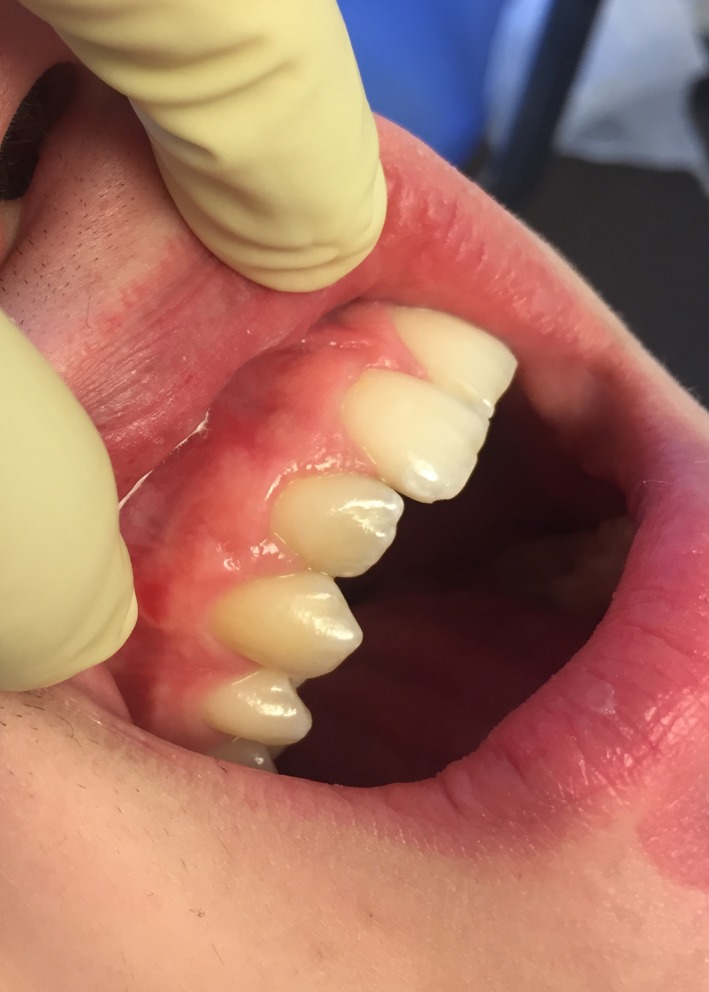
Clinical photography of the case in 24‐month follow‐up shows stable esthetic and functional conditions of the tooth #12

**Figure 5:**
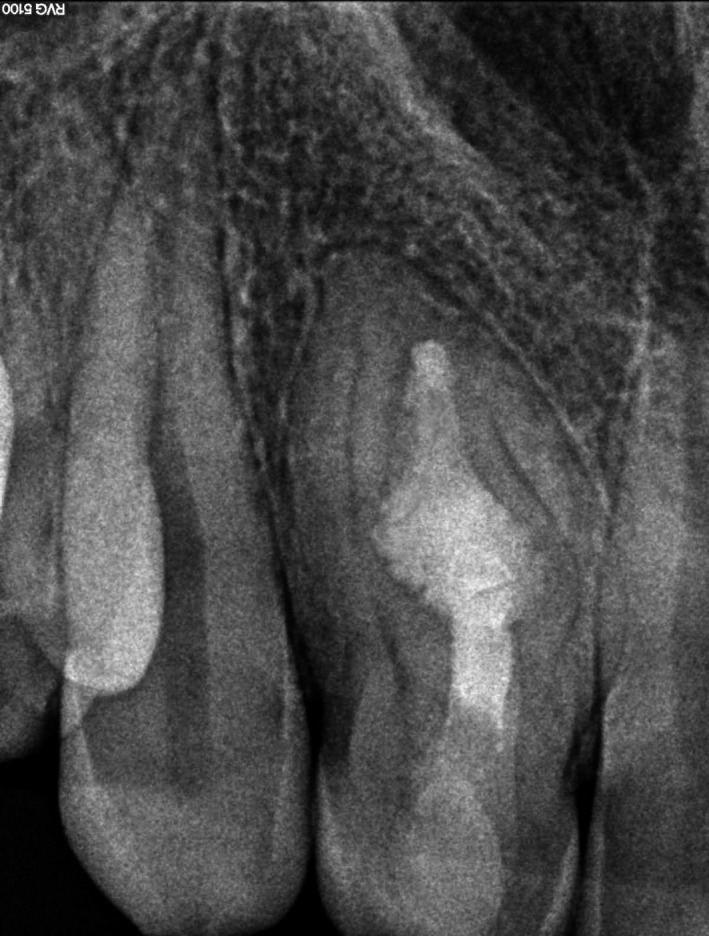
Radiographic image of tooth #12 in 24‐month follow‐up

**Figure 6:**
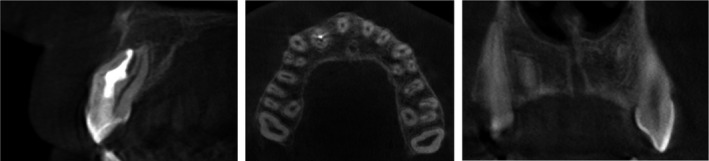
Cone‐beam computed tomographic (CBCT) imaging in 12‐month follow‐up shows successful healing of bone structures

**Figure 7:**
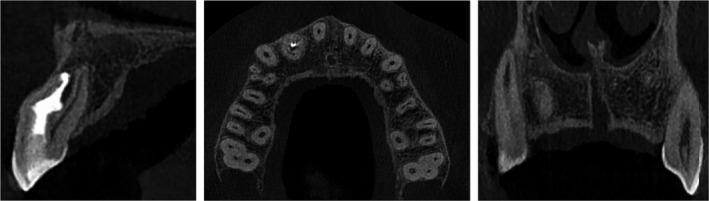
Cone‐beam computed tomographic (CBCT) imaging in 24‐month follow‐up shows total reparation of bone structures

The follow‐up visits of the patient were made after 1, 2, 3, 6, 12, and 24 months. Tooth #12 maintained a normal response to cold test for the entire period. The clinical examination revealed normal function of tooth #12 (Figure 4). The patient had no complaints or signs of infection. After 12 months, the radiographic examination showed a fully repaired bone structure (Figures 5, 6, 7).

## 3 DISCUSSION

Clinical management of teeth with infected type 3 invagination and PIP is a real challenge for clinicians. Based on the complex dental anatomy, different treatment models may be suggested, including nonsurgical endodontic treatment of the infected invagination,^8^, ^9^, ^10^ treatment of entire root canal system,^11^, ^12^, ^13^ combined endodontic and surgical treatment,^14^, ^15^, ^16^ or regenerative endodontic procedures.^17^, ^18^ In our case, no previously suggested treatment methods fitted the situation. We had to apply a combined treatment model in order to preserve the vital pulp, and the root structure, to treat invagination and to remove cystic lession.

A 3‐dimensional picture of the invaginated tooth can be very helpful in the process of treatment planning.^6^ CBCT imaging supplies a precise simulation of invagination as well as of the real canal, the periapical area, and external morphology in all dimensions. In the described case, the CBCT scan was also useful for demonstration of how the invagination compressed and distorted the pulp space at different levels although did not communicate with the regular canal.

The positive response of tooth #12 to cold test as well as the signs of blood supply in the pulp horns (observed in the endo cavity under dental microscope although the thin dentin layer) led us predict that the pulp in the main root canal was still viable. Due to the complex anatomy, it was considered to avoid, as much as possible, perforation of the main root canal or devitalization of the pulp while treating the invagination or damaging it retrogradically on surgical phase.^7^ According to the reports in the literature, the pulp spaces with such complex anatomy cannot be expected to be effectively debrided and adequately obturated.^19^, ^20^ Treating the regular canal space would have resulted in total dentin destruction of root with such morphology and probably would have led us to total clinical failure.

Disinfection of the DI was achieved by irrigation with copious amount of 2.5% NaOCl and the final rinse with 17% EDTA, owing to their antibacterial activity and reduction in the endodontic biofilms. Minimal hand instrumentation was made in the DI space. Rotary instrumentation within the invagination is not recommended because the surface may be covered by enamel and has an unpredictable shape with the possibility of the instrument fracture. K‐files have been routinely recommended for instrumentation of such cases.^9^, ^10^, ^14^, ^15^ Ultrasonic instrumentation and passive irrigation are highly recommended as well.^7^

For the apical barrier formation, MTA Angelus was used, due to its biocompactability, sealing abilities for open apices cases and relatively short setting time.^21^ To avoid the crown discoloration,^22^ only the apical seal was performed, and the remaining space of the invagination was filled with thermoplastic gutta‐percha and sealer. To ensure preservation of the vital pulp, the cavity was partly sealed with “Biodentine.” “Biodentine”is known for bioactivity and for being suitable as a pulp capping agent and obturation material.^23^, ^24^ Furthermore, it causes less crown discoloration than MTA.^25^

Guo et al^26^ suggested that CBCT images could provide a moderately accurate differentiation between cysts and granulomas. Thus, the probability of the diagnosis of true cyst was high when at least four of the six proposed diagnostic criteria for periapical lesions were present on CBCT. In our case, 5 diagnostic points as described by Guo were found: The lesion was located at the apex of the involved tooth, the lesion had a well‐defined cortical border, circular shape, radiolucent central structure, and the cortical plate was perforated. Pitcher et al^27^ suggested that if the volume of the lesion was more than 247 mm^3^, there was 80% probability of a cyst. In our case, the volume of the lesion also was more than 247 mm^3^. These findings fulfill the diagnosis of radicular cyst, which was confirmed by histological evaluation. Reported case confirms the advantages of CBCT use for periapical pathology differentiation.

Enucleation of the cystic lesion under the dental microscope resulted in high precision of the procedure, and prevented perforation of the sinus or, the nasal cavities, as well as unnecessary damage of the apical region of tooth #12. In the invagination space, root dentin was covered with impermeable enamel layer, so there were no lateral canals, apical seal was made with MTA, and the real canal space of the tooth was not infected. The possibility of reinfection into periapical area from the tooth was considered as very low, and no root resection or retrograde filling was indicated.

Preservation of the pulp vitality allowed us to save as much as possible of the root dentin and consequently increased our expectations for the long‐term treatment success. The extended follow‐up period of 24 months proved the successful outcome of the selected treatment procedures.

Thus, the presented case confirms a possibility to save a tooth with Oehlers Type 3 anomaly, with the preserved vital pulp, even when surgical cyst removal is performed. To our knowledge, no such cases have been described in the literature before.

## 4 CONCLUSION

In the presented case, endodontic treatment success was based on careful diagnostics and on adequate treatment planning. All efforts should aim to treat peri‐invaginated periodontitis and to preserve pulp vitality in Oehlers Type 3 anomalies.

## 5 IMPORTANCE

- All efforts should be aimed to safe permanent tooth for adolescents.
- Endodontic treatment success was based on careful diagnostics and on adequate treatment planning.
- Presented case confirms a possibility to save a tooth with Oehlers Type 3 anomaly and to preserve vitality of the tooth pulp, even when surgical cyst removal is performed.

## CONFLICT OF INTEREST

None declared.

## AUTHOR CONTRIBUTION

AD and RV: performed the treatment; AD: performed the follow‐up; and VM and RV: led the writing.
